# Cumulative triglyceride-glucose index is a risk for CVD: a prospective cohort study

**DOI:** 10.1186/s12933-022-01456-1

**Published:** 2022-02-10

**Authors:** Haozhe Cui, Qian Liu, Yuntao Wu, Liying Cao

**Affiliations:** 1 School of Medicine, Nankai University, Tianjin, China; 2 Department of Cardiology, Kailuan General Hospital, Tangshan, 063000 China; 3 Department of Hepatobiliary Surgery, Kailuan General Hospital, Tangshan, 063000 China

**Keywords:** Triglyceride-glucose index, Cardiovascular disease, Cumulative exposure, Cohort study, Prevention

## Abstract

**Background:**

Previous studies has shown a significant relationship between baseline triglyceride-glucose (TyG) index and cardiovascular disease (CVD). However, the long-term effect of TyG index and incident CVD remains uncertain. This study aimed to investigate the association between cumulative TyG index and the risk of CVD.

**Method:**

In this study, we recruited individuals participating in Kailuan Study from 2006 to 2013 without stroke, myocardial infarction, and cancer in the four consecutive examinations. Cumulative TyG index was calculated by multiplying the average TyG index and the time between the two consecutive examinations. Cumulative TyG index levels were categorized into four quartile groups: Q1 group, ≤ 50.65 (as reference group), Q2 group, 50.65–53.86, Q3 group, 53.86–57.44, Q4 group, > 57.44. The association between cumulative TyG index and the risk of CVD was estimated by multivariable Cox proportional hazard models.

**Result:**

A total of 44,064 individuals participated in the final analysis. After a mean follow-up of 6.52 ± 1.14 years, incident CVD, MI and stroke occurred in 2057, 395 and 1695, respectively. The risk of developing CVD increased with the quartile of cumulative in TyG index, after adjustment for multiple potential confounders, the HR for CVD events were 1.25 (1.08–1.44) in Q2, 1.22 (1.05–1.40) in Q3 and 1.39 (1.21–1.61) in Q4, compared to Q1 group. The longer duration of higher TyG index exposure was significantly associated with increased CVD risk. Similar results were obtained in the subgroup and sensitivity analysis.

**Conclusion:**

Cumulative TyG index was associated with increased risk of CVD. Maintaining an appropriate level of TG and FBG within the desirable range and better control of cumulative TyG index are important for prevention of CVD.

**Supplementary Information:**

The online version contains supplementary material available at 10.1186/s12933-022-01456-1.

## Introduction

According to epidemiological studies, cardiovascular disease (CVD) is still the leading cause of morbidity and mortality among chronic non-communicable diseases globally. Despite improvement in CVD outcomes following the progress of living standards and medical levels, the high incidence of CVD remains a major health concern [1]. Chronic conditions such as diabetes mellitus and hypertension are major modifiable risk factors contributing to the current epidemic of CVD [2, 3]. In addition, the burden of CVD is compounded further by unhealthy behavioral factors (including smoking, sedentary, obesity), and these risk factors also exacerbate the risk of mortality [4]. Thus, identifying effective preventive strategies for CVD has become a global public health priority.

Epidemiological and pathophysiological studies suggest that insulin resistance (IR) may be largely responsible for vascular endothelial dysfunction and CVD [5, 6]. The triglyceride-glucose (TyG) index is calculated using fasting blood glucose (FBG) and triglyceride (TG) levels, and it has been reported to be significantly correlated with IR and to be a simple and reliable surrogate marker of IR [7]. Moreover, several cohort studies have suggested that TyG index was a risk factor for development of CVD [8–10]. The TyG index is a dynamic condition that changes over time, but previous results might be due to the limited sample size and short follow-up periods, and there remains uncertainty about the associations with the cumulative TyG index and incident CVD. Those features do not take into account the exposure time of each TyG index and the risk that those features may convey.

Therefore, in the present study, we investigated to determine the impact of cumulative TyG index, a measure which incorporates both the levels of TyG index and the duration of the exposure elevated TyG index, on the risk of developing CVD by using a large community-based prospective cohort from Kailuan Study.

## Methods

### Study participants

**Fig. 1:**
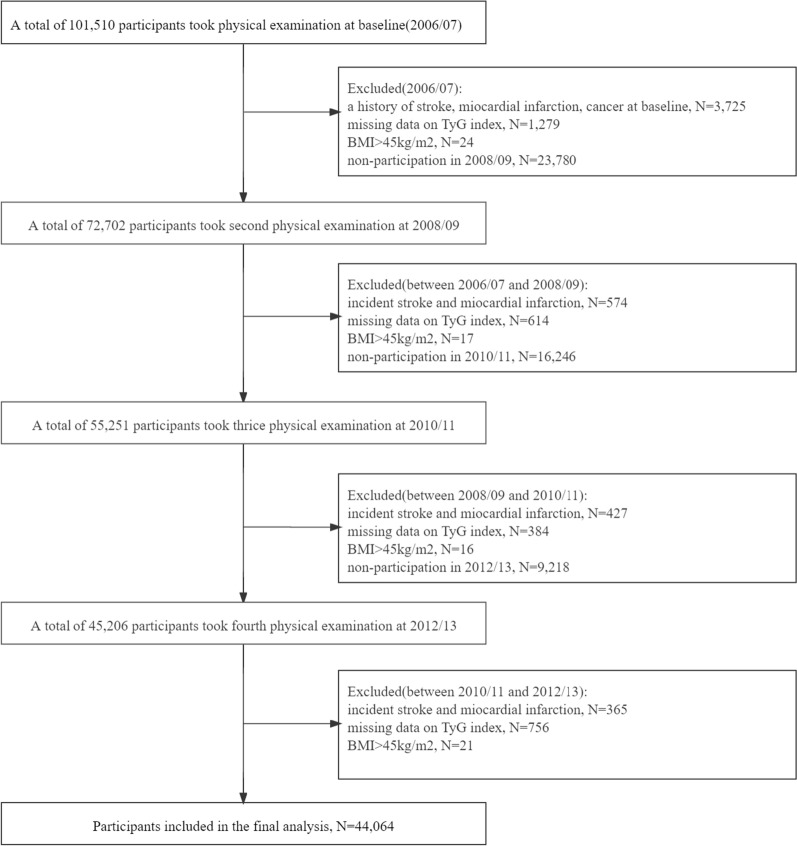
Flowchart of the study population

The Kailuan Study is an ongoing prospective community-based cohort study conducted in Tangshan, China. All participants in the Kailuan Study are employees and retirees of the Kailuan Group. Details of the study design and procedure have been described elsewhere [11]. At baseline, 101,510 participants (81,110 males and 20,400 females; aged 18–98 years) were recruited, underwent clinical and laboratory examinations, and completed a questionnaire interview (June 2006 to October 2007) at 11 hospitals affiliated with the Kailuan Group. Subsequent examinations involving anthropometric, cardiovascular risk factor measures, and self-reported questionnaires (including income, educational level, drinking and so on) occurred approximately biennially after baseline until December 2017 (2008/09, 2010/11, 2012/13, 2014/15 and 2016/17). For our investigation, participants were eligible if they attended three consecutive examinations between the second (2008/09), the third (2010/11) and the fourth (2012/13) examinations. Participants were excluded if they had prevalent myocardial infarction (MI), stroke or cancer, respectively, in or prior to 2012, or missing data on FBG or TG at any of the examinations, or BMI > 45 kg/m^2^ at any of the examinations. Ultimately, a total of 44,064 participants were enrolled in the present study (Fig. 1).

The study was conducted in accordance with the guidelines of the Declaration of Helsinki and was approved by the Kailuan General Hospital Ethics Committee. All the participants agreed to take part in the study and provided written informed consent.

### Data collection and definitions

Information on demographic and clinical characteristics (age, sex, lifestyle, and past medical history, etc.) were collected using a self-reported questionnaire, as detailed elsewhere [12]. Education level was classified as primary school or below, middle school, and high school or above. Smoking and drinking status were classified as yes or no. Active physical exercise was defined as “ > 4 times per week and 20 min at time”. BMI was calculated as the weigh (kg)/height^2^ (m^2^).

Elbow venous blood samples of 5 mL were collected into an anticoagulant tube containing EDTA between 7:00–9:00 am after overnight fasting for at least 8 h, and the serum was collected after centrifugation at 3000 × *g* for 10 min. The supernatant was measured within 4 h. All biochemical measurement including TG, high-density lipoprotein cholesterol (HDL-C), low-density lipoprotein cholesterol (LDL-C), high-sensitive C-reactive protein (Hs-CRP), FBG, and Uric acid (UA), and etc. was measured on the Hitachi 747 autoanalyzer (Hitachi, Tokyo, Japan).

Hypertension was defined as SBP ≥ 140 mmHg or DBP ≥ 90 mmHg, a self-reported history of hypertension, or any use of antihypertensive medication. Diabetes was defined as FBG ≥ 7.0 mmol/L, a self-reported history of diabetes, or use of antidiabetic medication.

### Cumulative TyG index

The TyG index was calculated as ln (fasting TG [mg/dL] × FBG [mg/dL]/2), as previously reported [13]. Cumulative TyG index was defined as the summation of average TyG index for each pair of consecutive examinations multiplied by the time between these two consecutive visits in years:

$$\left[ {\left( {{\text{TyG index}}_{{{2}00{6}}} \, + \,{\text{TyG index}}_{{{2}00{8}}} } \right)/{2}*{\text{time}}_{{{1} - {2}}} } \right]\, + \,\left[ {({\text{TyG index}}_{{{2}00{8}}} \, + \,{\text{TyG index}}_{{{2}0{1}0}} } \right)/{2}*{\text{time}}_{{{2} - {3}}} \left] {\, + \,} \right[({\text{TyG index}}_{{{2}0{1}0}} \, + \,{\text{TyG index}}_{{{2}0{12}}} )/{2}*{\text{time}}_{{{3} - {4}}} ]$$

where TyG index_2006_, TyG index_2008_, TyG index_2010_, and TyG index_2012_ indicate the TyG index at the baseline, second, third and fourth examinations, and time_1-2_, time_2-3_ and time_3-4_ indicate the participant-specific time intervals between consecutive visits in years. The means of time_1-2_, time_2-3_ and time_3-4_ were 2.06 years, 1.95 years and 2.22 years. The participants were stratified by quartile of cumulative TyG index: Q1 group, ≤ 50.65 (as reference group), Q2 group, 50.65–53.86, Q3 group, 53.86–57.44, Q4 group, > 57.44.

According to previous studies, adults with a higher TyG index experience an increased risk of CVD, in the current analysis, participants with a fourth quartile of TyG index (> 9.02) at each examination were identified to be in the higher TyG index exposure group [9]. TyG index exposure duration was defined as the times of examinations with higher TyG index among the 4 examinations, quantified as 0 years (never had higher TyG index), 2 years (had higher TyG index once), 4 years (had higher TyG index twice), 6 years (had higher TyG index thrice), and 8 years (had higher TyG index at all 4 study examinations).

### Assessment of CVD

Follow-up ended at the first record of CVD event, all-cause death or at the end of follow-up on 31 December 2019, whichever came first. The types of CVD included MI and stroke. We used ICD-10th revision codes to identify CVD cases (I21 for MI, I6 for stroke) [14, 15]. The database of CVD diagnoses was obtained from the Municipal Social Insurance Institution and Hospital Discharge Register and was updated annually during the follow-up period. An expert panel collected and reviewed annual discharges records from 11 local hospitals to identify patients who were suspected of CVD. The diagnosis of MI was determined by the patient’s clinical symptoms, electrocardiogram, and dynamic changes of myocardial enzyme following the World Health Organization’s Multinational Monitoring of Trends and Determinants in Cardiovascular Disease criteria [16]. Stroke was diagnosed based on neurological signs, clinical symptoms, and neuroimaging tests, including computed tomographic or magnetic resonance imaging, in line with the World Health Organization criteria [17].

### Statistical analysis

Continuous variables were compared using analysis of variance or the Kruskal–Wallis test according to distribution, and categorical variables were compared with the chi-square test.

Kaplan–Meier method was used to compute cumulative incidence of CVD and subgroup of CVD. Cox proportional hazard models were used with age as the time scale to estimate the hazard ratios (HRs) for incident CVD by cumulative TyG index, and were adjusted for baseline confounders, including sex, income (categories of high, intermediate, and low), educational level, drinking (yes or no), smoking (yes or no), physical exercise (active or inactive), diabetes, hypertension, lipid‐lowering medication, BMI, resting heart rate(RHR), HDL-C, LDL-C, UA, Hs-CRP. Missing covariates were imputed by multiple imputation using the fully conditional specification method SAS MI procedure. The results were consistent from analyses that excluded participants with missing covariates. The proportional hazard assumption was examined by Schoenfeld residuals.

To examine the robustness of our results, we performed several sensitivity analyses. First, we excluded events occurring in the first 2 years of follow-up to minimize potential reverse causation. Second, we excluded participants with diabetes or received treatment with lipid lowering medication and repeated analysis. Third, additional adjustment for TyG index at baseline. All analyses were done with SAS 9.4 (SAS Institute, Cary, NC), at a two-tailed alpha level of 0.05.

## Results

### Baseline characteristics of participants

A total of 44,064 eligible participants were included in present analysis, their mean age was 54.30 ± 11.65 years, and 74.80% were men. The average cumulative TyG index was 54.12 ± 5.13. To baseline characteristics of participants according to the quartile of cumulative TyG index are shown in Table 1. When compared to the Q1 group, participants in the other groups were more likely to be older, men, less well educated, more current smokers and drinkers, a higher prevalence of hypertension, diabetes, and lipid‐lowering medication, more likely to take antihypertensive agents and antidiabetic agents, had a higher BMI, RHR, LDL-C, UA and Hs-CRP level, and a lower HDL-C level.

**Table 1:** Baseline characteristics of participants by cumulative TyG index quartiles (n = 44064)

	Q1 group	Q2 group	Q3 group	Q4 group	*P*
N	11,016	11,017	11,017	11,014	< 0.001
Age, mean ± SD, years	49.92 ± 10.69	52.78 ± 11.43	56.25 ± 11.47	58.22 ± 11.21	< 0.001
Male, %	7670 (69.63)	8431 (76.53)	8527 (77.40)	8328 (75.61)	< 0.001
BMI, mean ± SD, kg/m^2^	24.15 ± 3.19	24.77 ± 3.24	25.28 ± 3.24	25.87 ± 3.18	< 0.001
RHR, mean ± SD, beats/min	71.66 ± 9.73	72.83 ± 10.54	73.78 ± 10.56	75.16 ± 10.67	< 0.001
HDL-C, mmol/L	1.43 (1.21–1.69)	1.38 (1.19–1.60)	1.32 (1.11–1.55)	1.20 (0.99–1.44)	< 0.001
LDL-C, mmol/L	2.24 (1.68–2.84)	2.50 (2.01–2.97)	2.50 (2.00–3.05)	2.59 (2.07–3.13)	< 0.001
UA, mean ± SD, μmol/L	307.27 ± 91.66	296.64 ± 92.90	309.76 ± 89.45	326.33 ± 89.92	< 0.001
Hs-CRP, mg/dL	1.12 (0.39–2.97)	0.90 (0.20–2.19)	1.23 (0.51–2.63)	1.59 (0.83–3.00)	< 0.001
High income, %	754 (23.20)	746 (22.95)	837 (25.75)	913 (28.09)	< 0.001
High school or above, %	1061 (28.85)	939 (25.53)	871 (23.68)	807 (21.94)	< 0.001
Drinking, %	1625 (21.12)	1774 (23.06)	2035 (26.45)	2260 (29.37)	< 0.001
Smoking, %	3168 (23.85)	3085 (23.22)	3485 (26.23)	3546 (26.69)	< 0.001
Physical exercise, %	1066 (17.32)	1327 (21.56)	1821 (29.59)	1940 (31.52)	< 0.001
Diabetes, %	225 (6.78)	496 (14.94)	812 (24.46)	1787 (53.83)	< 0.001
Hypertension, %	2742 (16.49)	4180 (25.14)	4485 (26.97)	5220 (31.39)	< 0.001
Lipid‐lowering medication, %	245 (16.20)	288 (19.05)	415 (27.45)	564 (37.30)	< 0.001
TyG index_2006_, mean ± SD	8.18 ± 0.53	8.50 ± 0.57	8.71 ± 0.59	9.15 ± 0.67	< 0.001
TyG index_2008_, mean ± SD,	8.17 ± 0.51	8.52 ± 0.51	8.72 ± 0.54	9.24 ± 0.69	< 0.001
TyG index_2010_, mean ± SD	8.24 ± 0.50	8.52 ± 0.52	8.74 ± 0.54	9.27 ± 0.67	< 0.001
TyG index_2012_, mean ± SD	8.29 ± 0.53	8.52 ± 0.55	8.74 ± 0.60	9.22 ± 0.71	< 0.001

*BMI* body mass index, *RHR* resting heart rate, *HDL* high-density lipoprotein, *LDL* low-density lipoprotein, *UA* uric acid, *Hs-CRP* high-sensitivity C-reactive protein, *TG* triglyceride, *FBG* fasting blood glucose, *TyG* triglyceride glucose

### Association between cumulative TyG index and risk of CVD

After a mean follow-up of 6.52 ± 1.14 years starting after the fourth examination, incident CVD, MI and stroke occurred in 2057, 395 and 1695, respectively. The Kaplan–Meier curve indicates a stepwise increase in the incidence of CVD across the patterns of cumulative TyG index (χ^2^ = 223.71, *P* < 0.001). The Kaplan–Meier incidence rates of MI and stroke are shown in Additional file 1: Fig. S1.  Table 2 reports the incidence rates of CVD and subtypes multivariable HR by categories of cumulative TyG index. Compared with those with Q1 group, risk of CVD was significantly higher in those with Q2 group (HR 1.25; 95% CI 1.08, 1.44), Q3 group (HR 1.22; 95% CI 1.05, 1.40), and Q4 group (HR 1.39; 1.21, 1.61). Similar results were obtained for the subgroup analysis (Table 3).

**Table 2:** Association of cumulative TyG index with CVD from 2013 to 2019 (n = 44,064)

	Case	Cumulative incidence, %	Sex-adjusted	Multiple-adjusted
*Quartiles*				
Q1	307	3.13	Ref	Ref
Q2	474	4.97	1.36 (1.18–1.57)	1.25 (1.08–1.44)
Q3	544	5.89	1.39 (1.20–1.60)	1.22 (1.05–1.40)
Q4	709	7.51	1.78 (1.55–2.04)	1.39 (1.21–1.61)
P for trend	< 0.001			
Per SD	1.10 (1.05–1.16)			
*Time exposure duration*				
0 year	819	4.22	Ref	Ref
2 years	428	6.01	1.44 (1.28–1.62)	1.28 (1.14–1.44)
4 years	283	6.06	1.52 (1.33–1.74)	1.24 (1.08–1.43)
6 years	228	6.24	1.55 (1.34–1.80)	1.21 (1.04–1.42)
8 years	276	8.01	1.94 (1.69–2.22)	1.42 (1.22–1.66)
P for trend	< 0.001			

Multivariable model adjusted for sex, income, educational level, drinking, smoking, diabetes, hypertension, lipid‐lowering medication, BMI, RHR, HDL-C, LDL-C, UA, Hs-CRP

Culumative TyG index SD = 5.13

**Table 3:** Association of cumulative TyG index with subgroup of CVD from 2013 to 2019 (n = 44,064)

	MI			Stroke		
	Case	Cumulative incidence, %	Multiple-adjusted	Case	Cumulative incidence, %	Multiple-adjusted
*Quartiles*						
Q1	51	0.50	Ref	259	2.70	Ref
Q2	93	0.92	1.49 (1.05–2.10)	388	4.14	1.20 (1.02–1.40)
Q3	95	0.95	1.25 (0.89–1.78	455	5.01	1.19 (1.02–1.40)
Q4	138	1.40	1.53 (1.09–2.16)	584	6.26	1.35 (1.16–1.58)
P for trend	< 0.001			< 0.001		
Per SD	1.08 (0.97–1.21)			1.10 (1.05–1.16)		
*Time exposure duration*						
0 year	132	0.67	Ref	696	3.61	Ref
2 years	86	1.10	1.57 (1.19–2.07)	351	5.11	1.23 (1.08–1.40)
4 years	50	1.00	1.31 (0.94–1.84)	238	5.17	1.22 (1.05–1.43)
6 years	46	1.20	1.47 (1.03–2.10)	183	5.07	1.14 (0.96–1.36)
8 years	63	1.78	1.89 (1.34–2.65)	218	6.40	1.32 (1.12–1.57)
P for trend	< 0.001			< 0.001		

Multivariable model adjusted for sex, income, educational level, drinking, smoking, diabetes, hypertension, lipid‐lowering medication, BMI, RHR, HDL-C, LDL-C, UA, Hs-CRP

### Time exposure duration of TyG index and risk of CVD

**Fig. 2:**
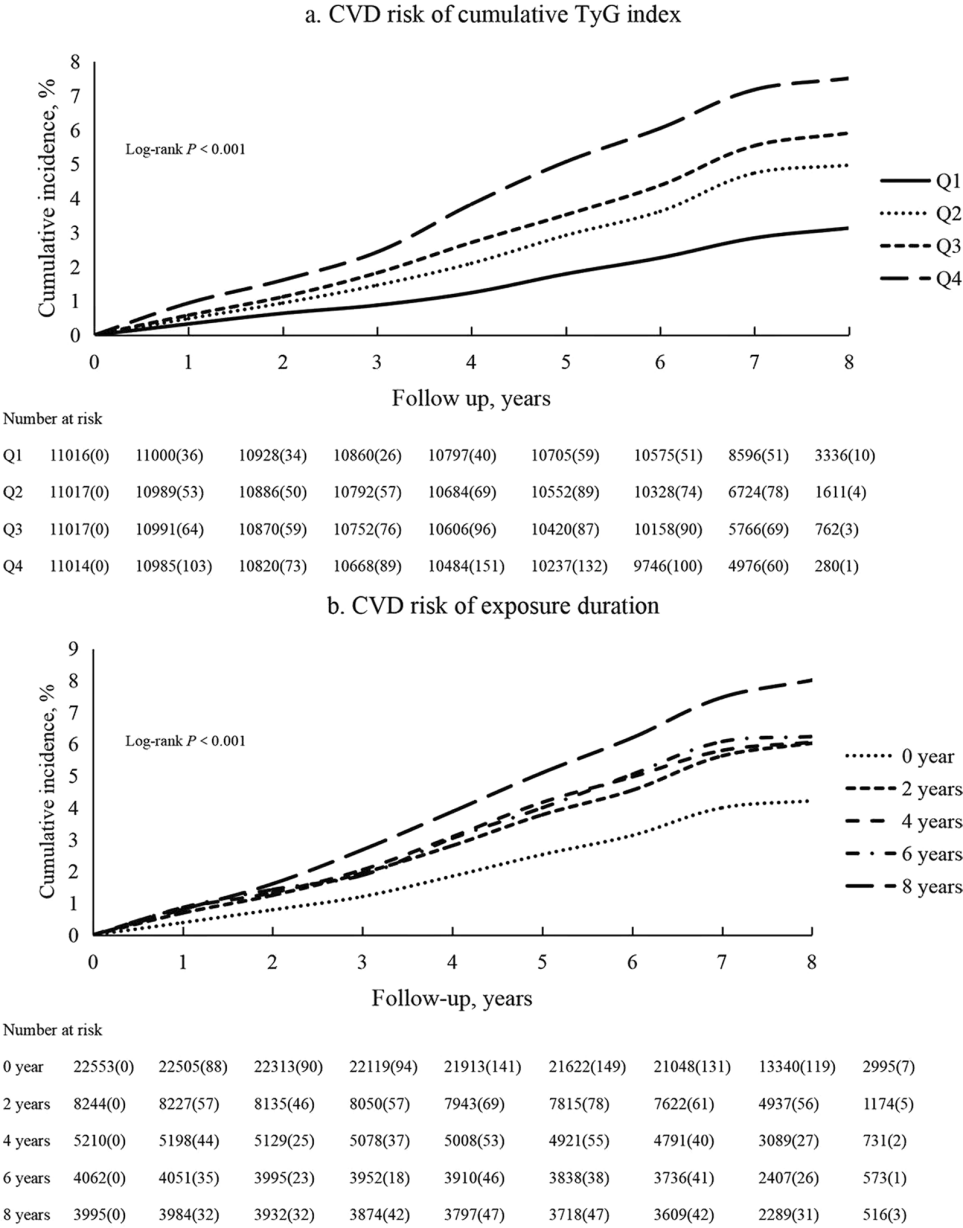
Kaplan–Meier incidence rate of CVD by TyG index. **a** Quartiles of cumulative TyG index. **b** Exposure duration with a higher TyG index

Figure 2b shows the Kaplan–Meier incidence rate stratified by the times of examinations with a higher TyG index. There was a progressively increasing risk of incidence with the times of examinations with a higher TyG index (P < 0.001). After adjustment for potential confounders, compared with the unexposed group (0 years), risk of CVD was significantly higher in those with 2 years group (HR 1.29; 95% CI 1.15, 1.46), 4 years group (HR 1.25; 95% CI 1.09, 1.44), 6 years group (HR 1.21; 95% CI 1.04, 1.42), and 8 years group (HR 1.42; 95% CI 1.22, 1.66) (Table 2).

### Sensitivity analysis

In the sensitivity analyses, the associations of cumulative TyG index with risk of incident CVD were not materially changed after excluding participants with CVD occurring within the first two years of the follow-up, or excluding participants who received treatment with lipid-lowering medications, or excluding participants with diabetes, or additional adjustment for TyG index at baseline (Table 4).

**Table 4:** Association of cumulative TyG index with CVD-sensitivity analysis

	Analysis 1			Analysis 2			Analysis 3			Analysis 4		
	CVD	MI	Stoke	CVD	MI	Stoke	CVD	MI	Stoke	CVD	MI	Stoke
*Quartiles*												
Q1	Ref	Ref	Ref	Ref	Ref	Ref	Ref	Ref	Ref	Ref	Ref	Ref
Q2	1.26 (1.07–1.49)	1.49 (0.99–2.23)	1.23 (1.02–1.47)	1.29 (1.11–1.50)	1.53 (1.07–2.19)	1.22 (1.03–1.43)	1.30 (1.12–1.52)	1.35 (0.95–1.92)	1.24 (1.05–1.47)	1.20 (1.04–1.40)	1.38 (0.98–1.96)	1.16 (0.99–1.36)
Q3	1.20 (1.02–1.42)	1.19 (0.79–1.80)	1.21 (1.01–1.44)	1.27 (1.09–1.47)	1.31 (0.91–1.88)	1.21 (1.03–1.42)	1.27 (1.09–1.48)	1.07 (0.74–1.54)	1.23 (1.04–1.45)	1.14 (0.99–1.33)	1.12 (0.78–1.60)	1.14 (0.94–1.34)
Q4	1.35 (1.15–1.59)	1.34 (0.89–2.02)	1.36 (1.14–1.63)	1.43 (1.23–1.66)	1.60 (1.11–2.29)	1.33 (1.13–1.57)	1.52 (1.31–1.77)	1.34 (0.94–1.91)	1.40 (1.19–1.66)	1.25 (1.06–1.46)	1.24 (0.85–1.80)	1.24 (1.04–1.47)
*Time exposure duration*												
0 year	Ref	Ref	Ref	Ref	Ref	Ref	Ref	Ref	Ref	–	–	–
2 years	1.28 (1.12–1.47)	1.46 (1.04–2.04)	1.26 (1.09–1.45)	1.22 (1.07–1.38)	1.51 (1.13–2.01)	1.16 (1.01–1.33)	1.24 (1.09–1.41)	1.51 (1.13–2.02)	1.20 (1.05–1.38)	–	–	–
4 years	1.26 (1.08–1.48)	1.22 (0.81–1.85)	1.27 (1.06–1.51)	1.23 (1.06–1.43)	1.24 (0.86–1.79)	1.23 (1.01–1.45)	1.19 (1.02–1.39)	1.21 (0.84–1.74)	1.19 (1.01–1.40)	–	–	–
6 years	1.24 (1.04–1.48)	1.54 (1.01–2.34)	1.18 (0.96–1.43)	1.10 (0.92–1.31)	1.32 (0.88–1.97)	1.05 (0.86–1.28)	1.18 (0.99–1.39)	1.26 (0.85–1.87)	1.15 (0.95–1.38)	–	–	–
8 years	1.51 (1.26–1.79)	2.08 (1.39–3.10)	1.42 (1.17–1.72)	1.42 (1.20–1.69)	2.11 (1.46–3.03)	1.29 (1.06–1.57)	1.46 (1.24–1.71)	1.78 (1.23–2.57)	1.37 (1.15–1.65)	–	–	–

Multivariable model adjusted for sex, income, educational level, drinking, smoking, diabetes, hypertension, lipid‐lowering medication, BMI, RHR, HDL-C, LDL-C, UA, Hs-CRP

Analysis1 excluded participants events occurring in the first 2 years of follow-up (n = 884); Analysis2 excluded participants with diabetes at baseline (n = 3320); Analysis3 excluded received treatment with lipid lowering medication at baseline and follow-up (n = 5866); Analysis4 additional adjustment for TyG index at baseline

## Discussion

In this prospective cohort study of 44,064 individuals from Kailuan study, we found an association between cumulative TyG index and the risk of CVD. In particular, higher exposure to cumulative TyG index was associated with CVD and subgroup of CVD in a period of mean 6.52 years. We also found that longer time of higher TyG index exposure could increase risk for CVD and subgroup. Additionally, this result was also validated in non-diabetes and not taking lipid-lowering medication populations.

Previous studies have found that participants with a higher TyG index were at a higher risk of developing CVD compared to low-level group [8–10]. However, in most studies, TyG index was based on single assessments, and ignoring the changes of TyG index over time, which would result in potential regression dilution bias and may affect the accuracy of the results. TyG index is calculated by TG and FBG, and these makers show dynamic changes in state. Therefore, the evaluation of the TyG index at baseline alone does not reflect the longitudinal association between the cumulative TyG index and CVD risk over time. In the present study, we found that cumulative TyG index had an increased risk of developing CVD. The risk of CVD development was highest in individuals with highest quartile group, with multivariate-adjusted HR of 1.39 (95% CI, 1.21–1.61). In addition, this risk was not attenuated by additional adjustment for baseline TyG index. The cumulative effect seems to be independent of- and better than the baseline TyG index in the pathogenesis of CVD. Likewise, the longest exposure time was associated with the highest risk of CVD.

Another important finding was derived from our subgroup analysis. Our findings indicate that cumulative TyG index seems to significantly increase the risk of developing MI and stroke, and the risk of cumulative TyG index developing in MI is slightly higher than that in stroke. Epidemiological survey showed that the prevalence of stroke patients are significantly more than patients with MI [18]. Although dyslipidemia and diabetes were observed to be risk factors for development of MI and stroke, these differences in the characteristics of disease itself create the potential for bias. Similarly, because of the limitations of case number, this may, in part, account for not reaching statistical significance in sensitivity analysis after excluding participants with outcomes occurring within the first 2 years of the follow-up.

To the best of our knowledge, few cohort studies have explored TyG index by repeated measurements analysis. Wang et al. found that higher levels of TyG index were associated with an increased risk of future stroke and ischemic stroke by updated cumulative average exposure [19]. In the Rural Chinese Study of a large prospective cohort of 5706 participants, the differences in TyG index between baseline and a subsequent examination was used to predict the risk of type 2 diabetes, and the results showed that the risk of incident diabetes was increased with the highest quartile of change in TyG index in normal-weight people [20]. Nevertheless, the relationship between cumulative exposure of TyG index and CVD has not been investigated in previous studies. The present findings imply that a substantial increase in TyG index is associated with a high risk of CVD, which highlights the importance of early controlling the glucose and lipids in clinical practice. Meanwhile, a multicenter cohort study is needed to further validate our results.

The increased risks of CVD and subgroup is possibly explained by persistent, low-degree inflammation, arterial stiffness and endothelial dysfunction caused by cumulative TyG index [21–23]. Previous studies have detected a positive association between arterial stiffness and TyG index as well as insulin resistance [24]. Continuous elevated of TyG index will lead to structural and functional changes in the arterial walls, resulting in further decrease of large arterial elasticity, and these changes could lead to CVD onset.

Our study has important implications for CVD prevention. The cumulative TyG index might help identify individuals at high risk for developing CVD in a large and long-term follow-up cohort. For the general populations, maintaining an appropriate level of TG and FBG within the desirable range and better control of cumulative TyG index is important for controlling the chronic diseases. For patients with diabetes and hyperlipidemia, motivating patients to adhere to glucose-lowering, and lipid-lowering medications and treatment monitoring for a long time for actively controlling FBG and TG levels [25, 26]. Strengths of the current study include the use of its prospective cohort design, large sample size, a long follow-up for CVD events and repeated measurements of multiple laboratory variables. However, our investigation has several limitations. First, serum insulin level was not directly collected and HOMA-IR could not be evaluated. In addition, HbA1C and 2 h-oral glucose tolerance test were not measured but was estimated by fasting blood sample that might have overestimated or underestimated the actual prevalence of diabetes. Finally, this was an observational study, and causality cannot be demonstrated.

## Conclusion

In the present study, we observed that cumulative TyG index was associated with increased risk of CVD. Hence, clinicians should consider the risk of incident CVD in people with abnormal FBG and TG and counsel them about metabolic fitness.

## Supplementary Information

**Additional file 1: Fig. S1.** Kaplan-Meier incidence rate of stroke and MI by TyG index. **a** quartiles of cumulative TyG index for stroke. **b** quartiles of cumulative TyG index for MI. **c** exposure duration with a higher TyG index for stroke. **d** exposure duration with a higher TyG index for MI.

## Data Availability

The datasets used and/or analyzed during the current study are available from the corresponding author on reasonable request.

## Abbreviations

BMI: Body mass index
CI: Confidence interval
CVD: Cardiovascular disease
FBG: Fasting blood glucose
HR: Hazard ratio
Hs-CRP: High-sensitivity C-reactive protein
LDL-C: Low-density lipoprotein cholesterol
SD: Standard deviation
TG: Triglyceride
TyG: Triglyceride-glucose
UA: Uric acid

## References

[1] Dagenais GR, Leong DP, Rangarajan S, Lanas F, Lopez-Jaramillo P, Gupta R (2020). Variations in common diseases, hospital admissions, and deaths in middle-aged adults in 21 countries from five continents (PURE): a prospective cohort study. Lancet.

[2] Roth GA, Mensah GA, Johnson CO, Addolorato G, Ammirati E, Baddour LM (2020). Global burden of cardiovascular diseases and risk factors, 1990–2019: update from the GBD 2019 study. J Am Coll Cardiol.

[3] Mosenzon O, Alguwaihes A, Leon JLA, Bayram F, Darmon P, Davis TME (2021). CAPTURE: a multinational, cross-sectional study of cardiovascular disease prevalence in adults with type 2 diabetes across 13 countries. Cardiovasc Diabetol.

[4] Younus A, Aneni EC, Spatz ES, Osondu CU, Roberson L, Ogunmoroti O (2016). A systematic review of the prevalence and outcomes of ideal cardiovascular health in US and non-US populations. Mayo Clin Proc.

[5] Bornfeldt KE, Tabas I (2011). Insulin resistance, hyperglycemia, and atherosclerosis. Cell Metab.

[6] Ma X, Dong L, Shao Q, Cheng Y, Lv S, Sun Y (2020). Triglyceride glucose index for predicting cardiovascular outcomes after percutaneous coronary intervention in patients with type 2 diabetes mellitus and acute coronary syndrome. Cardiovasc Diabetol.

[7] Hong S, Han K, Park CY (2020). The triglyceride glucose index is a simple and low-cost marker associated with atherosclerotic cardiovascular disease: a population-based study. BMC Med.

[8] Li S, Guo B, Chen H, Shi Z, Li Y, Tian Q (2019). The role of the triglyceride (triacylglycerol) glucose index in the development of cardiovascular events: a retrospective cohort analysis. Sci Rep.

[9] Barzegar N, Tohidi M, Hasheminia M, Azizi F, Hadaegh F (2020). The impact of triglyceride-glucose index on incident cardiovascular events during 16 years of follow-up: Tehran lipid and glucose study. Cardiovasc Diabetol.

[10] Alizargar J, Bai CH, Hsieh NC, Wu SV (2020). Use of the triglyceride-glucose index (TyG) in cardiovascular disease patients. Cardiovasc Diabetol.

[11] Ma X, Cui H, Sun M, Liu Q, Liu X, Li G (2021). Fasting blood glucose, cholesterol, and risk of primary liver cancer: the Kailuan study. Cancer Res Treat.

[12] Maoxiang Z, Lulu S, Lan S (2021). Associations of type 2 diabetes onset age with cardiovascular disease and mortality: the Kailuan study. Diabetes Care.

[13] Liu Q, Cui H, Ma Y, Han X, Cao Z, Wu Y (2021). Triglyceride-glucose index associated with the risk of cardiovascular disease: the Kailuan study. Endocrine.

[14] Jin C, Chen S, Vaidya A, Wu Y, Wu Z, Hu FB (2017). Longitudinal change in fasting blood glucose and myocardial infarction risk in a population without diabetes. Diabetes Care.

[15] Li W, Jin C, Vaidya A, Wu Y, Rexrode K, Zheng X (2017). Blood pressure trajectories and the risk of intracerebral hemorrhage and cerebral infarction: a prospective study. Hypertension.

[16] Tunstall-Pedoe H, Kuulasmaa K, Amouyel P, Arveiler D, Rajakangas AM, Pajak A (1994). Myocardial infarction and coronary deaths in the World Health Organization MONICA Project. Registration procedures, event rates, and case-fatality rates in 38 populations from 21 countries in four continents. Circulation.

[17] Goldstein M, Barnett M, Orgogozo JM, Sartorius N, Vereshchagin N (1989). Recommendations on stroke prevention, diagnosis, and therapy. Report of the WHO task force on stroke and other cerebrovascular disorders. Stroke.

[18] Peters SA, Yang L, Guo Y, Chen Y, Bian Z, Millwood IY (2017). Parenthood and the risk of cardiovascular diseases among 0.5 million men and women: findings from the China Kadoorie Biobank. Int J Epidemiol.

[19] Wang A, Tian X, Zuo Y, Chen S, Meng X, Wu S (2021). Change in triglyceride-glucose index predicts the risk of cardiovascular disease in the general population: a prospective cohort study. Cardiovasc Diabetol.

[20] Zhang M, Wang B, Liu Y, Sun X, Luo X, Wang C (2017). Cumulative increased risk of incident type 2 diabetes mellitus with increasing triglyceride glucose index in normal-weight people: the rural Chinese cohort study. Cardiovasc Diabetol.

[21] Festa A, Hanley AJ, Tracy RP, D'Agostino R, Haffner SM (2003). Inflammation in the prediabetic state is related to increased insulin resistance rather than decreased insulin secretion. Circulation.

[22] Janus A, Szahidewicz-Krupska E, Mazur G, Doroszko A (2016). Insulin resistance and endothelial dysfunction constitute a common therapeutic target in cardiometabolic disorders. Mediators Inflamm.

[23] Su Y, Wang S, Sun J, Zhang Y, Ma S, Li M (2021). Triglyceride glucose index associated with arterial stiffness in Chinese community-dwelling elderly. Front Cardiovasc Med.

[24] Adeva-Andany MM, Ameneiros-Rodríguez E, Fernández-Fernández C, Domínguez-Montero A, Funcasta-Calderón R (2019). Insulin resistance is associated with subclinical vascular disease in humans. World J Diabetes.

[25] Vinaya S (2020). Management of hypertriglyceridemia. BMJ.

[26] Hugh M, Damien L, Amanda A, Farmer A, Lewin I (2016). Management of type 2 diabetes in adults: summary of updated NICE guidance. BMJ.

