# Supplementary material for: Cumulative triglyceride-glucose index is a risk for CVD: a prospective cohort study
Source: Cardiovasc Diabetol. 2022 Feb 10;21:22. doi: 10.1186/s12933-022-01456-1 (PMC8830002; doi:10.1186/s12933-022-01456-1)
Supplement: Supplementary file 1 — Additional file 1: Fig. S1. Kaplan-Meier incidence rate of stroke and MI by TyG index. a quartiles of cumulative TyG index for stroke. b quartiles of cumulative TyG index for MI. c exposure duration with a higher TyG index for stroke. d exposure duration with a higher TyG index for MI. [file 12933_2022_1456_MOESM1_ESM.docx]

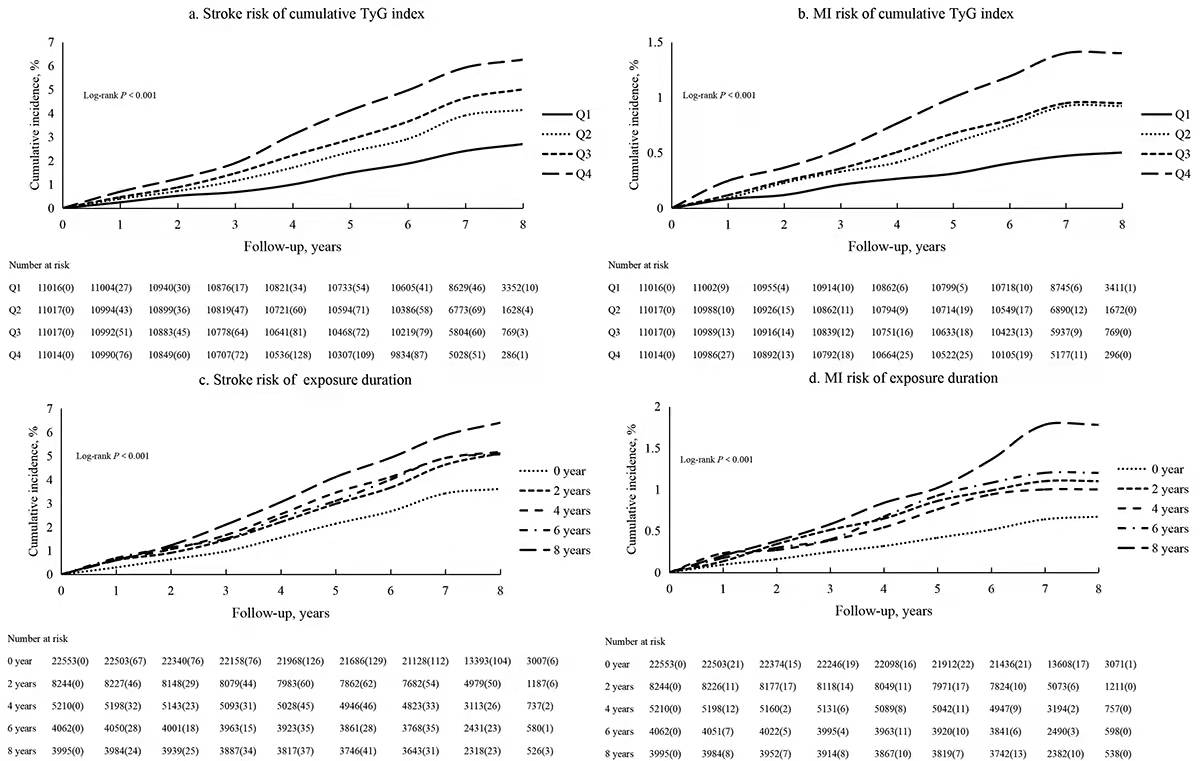


Fig. S1. Kaplan-Meier incidence rate of stroke and MI by TyG index(a quartiles of cumulative TyG index for stroke. b quartiles of cumulative TyG index for MI. c exposure duration with a higher TyG index for stroke. d exposure duration with a higher TyG index for MI).
